# Natural History of and Dynamic Changes in Clinical Manifestation, Serology, and Treatment of Brucellosis, China

**DOI:** 10.3201/eid2807.211766

**Published:** 2022-07

**Authors:** Hongyu Wang, Hongyan Liu, Qiran Zhang, Xiaobo Lu, Dan Li, Haocheng Zhang, Yan A. Wang, Rongjiong Zheng, Yi Zhang, Zhangfan Fu, Ke Lin, Chao Qiu, Yan O. Wang, Ye Gu, Jingwen Ai, Wenhong Zhang

**Affiliations:** National Medical Center for Infectious Disease, National Clinical Research Center for Aging and Medicine, Shanghai Key Laboratory of Infectious Diseases and Biosafety Emergency Response, Huashan Hospital, Fudan University, Shanghai, China (H. Wang, Q. Zhang, H. Zhang, Y. Zhang, Z. Fu, K. Lin, C. Qiu, J. Ai, W. Zhang);; The Sixth People’s Hospital of Shenyang, Shenyang, China (H. Liu, D. Li, Y.A. Wang, Y.O. Wang, Y. Gu);; Emergency Treatment and Innovation Center of Public Health Emergencies, Shenyang (H. Liu, D. Li, Y.A. Wang, Y.O. Wang, Y. Gu);; Center for Infectious Diseases, the First Affiliated Hospital of Xinjiang Medical University, Wulumuqi, China (X. Lu, R. Zheng);; Key Laboratory of Medical Molecular Virology (MOE/MOH), Shanghai Medical College, Fudan University, Shanghai (W. Zhang)

**Keywords:** brucellosis, Brucella, bacteria, zoonoses, dynamic surveillance, China

## Abstract

Serum agglutination test plus exposure history were used to diagnose most cases of human brucellosis in 2 China provinces. After appropriate treatment, 13.3% of acute brucellosis cases progressed to chronic disease; arthritis was an early predictor. Seropositivity can persist after symptoms disappear, which might cause physicians to subjectively extend therapeutic regimens.

## Abstract 2

In China, 13.3% of human brucellosis cases progressed to chronic disease; serum agglutination test might cause treatment elongation.

Brucellosis is a zoonosis caused by the bacterium *Brucella* that typically manifests in insidious onset of fever, malaise, arthralgias, and nonspecific physical findings, including hepatomegaly, splenomegaly, or lymphadenopathy (*1*). Accurate diagnosis and proper management of human brucellosis continues to challenge clinicians. Several studies have described the clinical characteristics of human brucellosis and evaluated diagnostic methods, but most of these studies are cross-sectional and focused on baseline manifestations or diagnostic accuracy (*2*–*6*). Much remains unclear about the dynamic changes of clinical manifestations, serology, and the tendency of brucellosis to persist and become chronic during development and treatment.

## The Study

We conducted a retrospective, real-world cohort study at 8 hospitals in Liaoning and Xinjiang Provinces, 2 of the most brucellosis-endemic areas in China, to investigate the characteristics of brucellosis during natural history and treatment. We enrolled patients confirmed to have brucellosis during 2014–2020. We collected information on contact history, clinical manifestations, laboratory parameters, and antibiotic therapy from the hospital information system and treatment outcome by telephone (Appendix). This research was carried out according to the principles of the Declaration of Helsinki. The study protocol was approved by the Ethics Committees of Huashan Hospital of Fudan University (KY2019–412). Informed consent was obtained from all patients before diagnosis, and patient data were anonymized.

We included 5,270 patients confirmed to have brucellosis during September 2014–December 2020. Three persons were excluded for positive HIV detection, 668 were excluded because they lacked positive culture or serologic results, and 1,191 were excluded for incomplete clinical information. We ultimately enrolled 3,411 persons; we performed follow-up for 1,676 persons at periods of 14, 28, 42, 90, 180, 360, or 720 days after diagnosis and treatment initiation (Appendix Figure 1).

Median participant age was 48 years (interquartile range 35.8–57.0 years). Most participants were men (2,452; 73.9%) and worked as farmers or herdsmen (2,776; 82.4%). A total of 2,066 (60.6%) had exposure history with suspicious animals, 1,686 (49.4%) had contact history with brucellosis patients, and 1,129 (33.1%) resided in a brucellosis-endemic area (Table 1).

**Table 1:** Demographic characteristics of brucellosis case-patients at enrollment in study of natural history and dynamic changes in clinical signs, serology, and treatment of brucellosis, China, 2014–2020*

Characteristic	Case-patients, n = 3,411
Median age, y (IQR)†	48.0 (35.8–57.0)
<20	143 (4.4)
20–40	933 (29.0)
40–60	1,629 (50.6)
>60	515 (16.0)
Sex‡	
M	2,452 (73.9)
F	867 (26.1%)
Nationality§	
Han	1,818 (53.6)
Others	1,572 (46.4)
Occupation¶	
Farmer	2,591 (76.9)
Herdsman	185 (5.5)
Veterinarian	33 (1.0)
Other	560 (16.6)
Contact history	
Exposure to suspicious animals	2,066 (60.6)
Contact with brucellosis patients	1,686 (49.4)
Residence in endemic area	1,129 (33.1)
Exposure to *Brucella*	58 (1.7)
Diagnostic test#	
Brucella culture	424 (23.2)
Antibody SAT	3,351 (99.1)
Titers**	
1:50	9 (0.4)
1:100	797 (35.2)
1:200	643 (28.4)
>1:400	815 (36.0)

*Values are no. (%) except as indicated. IQR, interquartile range; SAT, serum agglutination test. †Information on age was available for 3,220 participants. ‡Information on sex was available for 3,319 participants. §Information on nationality was available for 3,390 participants. ¶Information on occupation was available for 3,369 participants. #A total of 1,827 participants received *Brucella* culture, and 3,381 received SAT. **Among 3,381 participants tested by SAT, 2,264 had detailed positive titer information.

Blood cultures were collected from 1,827 participants for diagnostic purposes; results were positive in 424 (23.2%) persons. Serum agglutination tests (SAT) were collected from 3,381 persons, and 3,351 (99.1%) reported positive results. A total of 1,797 persons received both tests; 394 (21.9%) tested positive on both, 28 (1.6%) tested positive by culture only, and 1,375 (76.5%) tested positive by SAT only. Among 2,264 patients with positive titers on SAT, titers were >1:400 in 36.0%, 1:200 in 28.4%, 1:100 in 35.2%, and 1:50 in 0.4% (Table 1). Seasonal epidemics were observed during March–July each year, whereas total diagnosed cases decreased annually during 2015–2019 (Appendix Figure 2).

**Figure 1:**
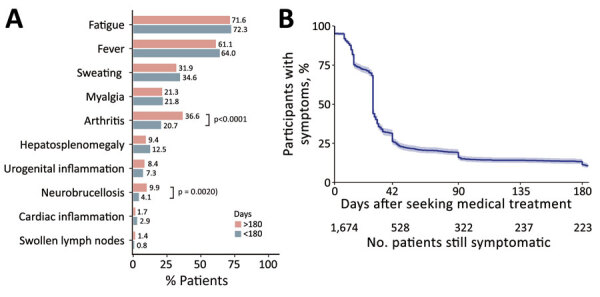
Dynamic characteristics of clinical manifestations in case-patients with acute and chronic brucellosis, China, 2014–2020. A) Natural symptom development with symptom duration <180 days (early stage) or >180 days (late stage) before patients received antibiotic therapy. B) Kaplan-Meier curve of symptomatic case-patients after treatment initiation.

We observed the natural history of brucellosis with symptom duration <180 days (early stage) or >180 days (late stage) before patients received antibiotic therapy. The 3 most common symptoms in early-stage disease were fatigue (72.3%), fever (64.0%), and sweating (34.6%). The most common symptoms in late-stage disease were fatigue (71.6%), fever (61.1%), and arthritis (36.6%) (Figure 1, panel A). Arthritis was more common in the late stage than the early stage (20.7%; p<0.0001). We observed neurobrucellosis in 9.9% of patients in the early stage and in 4.1% of patients in the late stage (p = 0.0020). After adjusting for confounding factors through propensity score-matching (PSM) (*7*), culture-diagnosed patients (compared with patients with SAT-diagnosed brucellosis) had higher incidence of fever (311 [81.8%] vs. 244 [58.9%]; p<0.0001), sweating (177 [46.6%] vs. 95 [25.0%]; p<0.0001), poor appetite (271 [71.3%] vs. 195 [51.3%]; p<0.0001), and hepatosplenomegaly (67 [17.6%] vs. 45 [11.8%]; p<0.0001). These patients also exhibited higher C-reactive protein (34.5 + 1.8 vs. 24.7 + 1.7; p = 0.0002) and erythrocyte sedimentation rate (45.6 + 1.7 vs. 29.3 + 1.4; p = 0.0290), which could be caused by active bloodstream infection (Appendix Table 1).

Among 1,676 participants with whom we conducted follow-up, we observed further clinical characteristics after treatment initiation. Most newly developed manifestations were reported within the first 2 weeks, but most patients recovered with persistent treatment (Appendix Figure 3). Two weeks after treatment initiation, 107 patients had newly developed cardiac inflammation, 112 neurobrucellosis, 140 urogenital inflammation, and 146 arthritis. Overall, 1,453 (86.7%) persons with acute brucellosis symptomatically recovered within 180 days after appropriate treatment, whereas 223 (13.3%) were still symptomatic after 180 days and chronic brucellosis developed (Figure 1, panel B) (*8*). In the chronic phase, arthritis (89 [25.6%]), fatigue (60 [17.3%]), and fever (57 [16.4%]) became the 3 most common manifestations (Appendix Figure 4).

After conducting PSM for age, sex, nationality, and year of enrollment, we performed multivariate logistic regression to identify risk factors for chronic brucellosis in 148 acute cases and 148 chronic cases (Table 2). Fever, sweating, myalgia, arthritis, and C-reactive protein and erythrocyte sedimentation rates at baseline were possible predictors for chronic brucellosis in univariate analysis (p<0.10). Arthritis was the only risk factor after multivariate analysis (odds ratio 4.11 [95% CI 1.22–16.92]; p = 0.0318).

**Table 2:** Comparison of acute and chronic brucellosis at enrollment in study of natural history and dynamic changes in clinical signs, serologic testing, and treatment of brucellosis, China, 2014–2020*

Characteristic	Acute brucellosis, n = 148	Chronic brucellosis, n = 148	Univariate analysis		Multivariate analysis

OR (95% CI)	p value	OR (95% CI)	p value
Symptom, no. (%)							
Fever	108 (73.0)	97 (65.5)	0.74 (0.53–1.04)	0.0753		1.28 (0.35–5.42)	0.7159
Sweating	52 (35.1)	65 (43.9)	1.38 (1.00–1.90)	0.0471		2.33 (0.69–7.55)	0.1578
Myalgia	30 (20.3)	45 (30.4)	1.75 (1.23–2.45)	0.0014		2.48 (0.68–8.34)	0.1466
Poor appetite	89 (60.1)	92 (62.2)	1.06 (0.77–1.47)	0.7427			
Hepatosplenomegaly	23 (15.5)	21 (14.2)	0.82 (0.50–1.28)	0.3971			
Arthritis	63 (42.6)	74 (50.0)	1.68 (1.22–2.31)	0.0013		4.11 (1.22–16.92)	0.0318
Urogenital inflammation	18 (12.2)	13 (8.8)	0.75 (0.43–1.24)	0.2883			
Neurobrucellosis	5 (3.4)	8 (5.4)	1.60 (0.76–3.06)	0.1805			
Laboratory test result, + SD							
Leukocytes, 10^9^ cells/L	6.4 + 0.2	5.9 + 0.2	0.96 (0.89–1.03)	0.2480			
Lymphocytes, 10^9^ cells/L	1.9 + 0.1	2.0 + 0.1	0.92 (0.75–1.12)	0.4038			
Monocytes, 10^9^ cells/L	0.5 + 0.1	0.5 + 0.0	0.68 (0.35–1.13)	0.2227			
CRP, mg/dL	33.7 + 3.6	23.8 + 3.3	0.99 (0.99–1.00)	0.0522		0.99 (0.97–1.02)	0.6948
Procalcitonin, ng/mL	0.1 + 0.0	0.2 + 0.1	1.02 (0.64–1.29)	0.8930			
ESR, mm/h	42.8 + 6.9	20.9 + 6.4	0.97 (0.95–1.00)	0.0481		0.98 (0.94–1.00)	0.1371

*CRP, C-reactive protein; ESR, erythrocyte sedimentation rate; OR, odds ratio.

**Figure 2:**
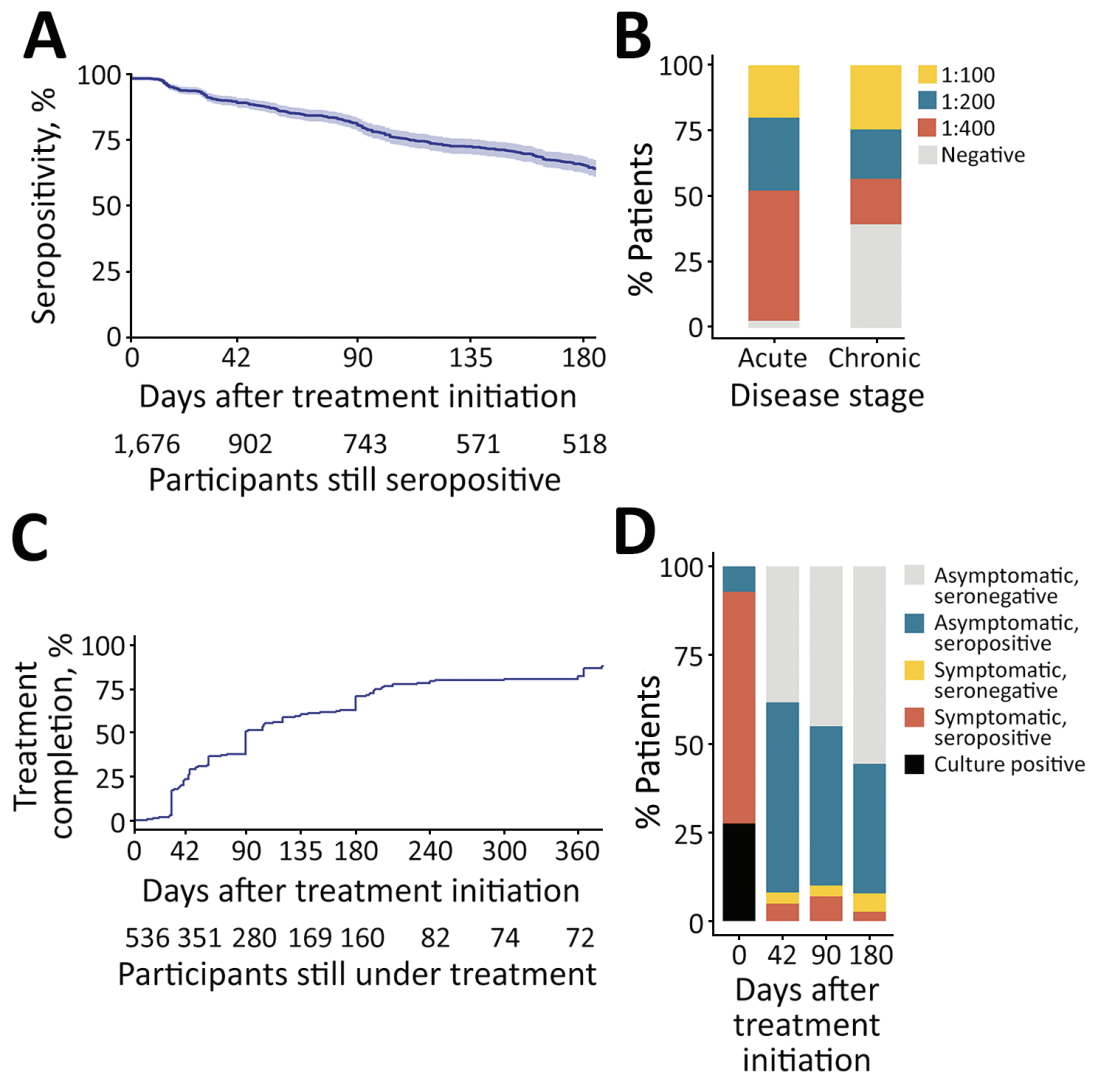
Dynamic characteristics of serum agglutination test and treatment courses in case-patients with brucellosis, China, 2014–2020. A) Seroconversion after treatment initiation; B) serum agglutination test titer distribution at baseline and 180 days after treatment initiation; C) treatment length of case-patients without systemic involvement; D) possible reasons for lengthened treatment in brucellosis case-patients without systemic involvement.

Dynamic SAT surveillance among 1,676 participants suggested that 53.8% (902/1,676) remained seropositive 42 days after treatment and 33.9% (518/1,676) remained seropositive 180 days after treatment (Figure 2, panel A). In acute cases, 413 remained seropositive and 1,040 seroconverted after 180 days. In chronic cases, 105 remained seropositive and 118 seroconverted (p<0.0001). The overall SAT titers decreased at the chronic phase; fewer patients had a titer of >1:400 (Figure 2, panel B).

We observed treatment outcomes in 432 patients without systemic involvement, of whom 307 (71.1%) received doxycycline and rifampin, 29 (6.7%) received doxycycline and levofloxacin, and 96 (22.2%) received triad therapy. In comparison with the standard 6-week treatment course (*8*–*10*), 75.2% (325/432) patients received antibiotic therapy for >42 days; median course of treatment was 90 (interquartile range 43–193) days (Figure 2, panel C). Further analysis in treatment elongation found that 26/325 (8.0%) were still symptomatic; the most common manifestations were sweating (61.5%), fatigue (50.0%), and fever (26.9%). A total of 174/325 (53.5%) participants were asymptomatic but seropositive, which could lead clinicians to subjectively extend antibiotic treatment; 125/325 (38.5%) participants were asymptomatic and seronegative (Figure 2, panel D). We further analyzed 107 participants who completed treatment within 42 days to determine whether standard treatment led to persistent symptoms or recurrence. Of those participants, 48/107 (44.9%) remained seropositive, 2/107 (1.9%) reported persistent symptoms, and 1/107 (0.9%) participant’s illness was considered a recurrence 2 years later.

The first limitation of our study is that we failed to follow up culture results during treatment. Second, we failed to distinguish transient and persistent exposure history, which might play a role in persistent symptoms or serologic results. Finally, infection was diagnosed by heterogenous methods, including culture and a series of serologic tests, which might introduce bias in baseline and prognosis analysis.

## Conclusions

Our study gives a thorough, dynamic description of clinical characteristics and serologic surveillance during the natural history and treatment of human brucellosis in a large population. Culture was 23.2% positive but SAT 99.1% positive in confirmed brucellosis. SAT plus exposure history remained the most effective diagnostic tool. Human brucellosis had variable manifestations at different disease stages. Untreated cases mainly manifested as fatigue, fever, or sweating in the early stage, whereas fatigue, fever, and arthritis were the most common symptoms at the late stage. After appropriate treatment, 13.3% of acute brucellosis cases progressed to chronic disease. Arthritis can serve as an early predictor of chronic brucellosis. Seropositivity can persist after symptoms disappear, which might cause physicians to subjectively and unnecessarily extend therapeutic regimens.

Appendix Additional information about natural history and dynamic changes in clinical manifestation, serology, and treatment of brucellosis, China
