# Supplementary material for: Natural History of and Dynamic Changes in Clinical Manifestation, Serology, and Treatment of Brucellosis, China
Source: Emerg Infect Dis. 2022 Jul;28(7):1460–5. doi: 10.3201/eid2807.211766 (PMC9239867; doi:10.3201/eid2807.211766)
Supplement: Appendix — Additional information about natural history and dynamic changes in clinical manifestation, serology, and treatment of brucellosis, China [file 21-1766-Techapp-s1.pdf]

Article DOI: https://doi.org/10.3201/eid2807.211766

Natural History and Dynamic Changes in Clinical Manifestation, Serology, and Treatment of Brucellosis, China

Appendix

Methods

Study Design

This is a multi-center, retrospective, real-world cohort study. Patients were recruited from September 2014 to December 2020 at 8 hospitals in Liaoning and Xinjiang Province, China, all of which are located in endemic areas of human brucellosis. All the patients enrolled were confirmed to be infected with brucellosis by meeting at least one of the following criteria according to the national guidelines (*1*): 1) positive culture of Brucella in blood or other sample; 2) positive ELISAs for IgG; 3) positive indirect Coomb's test with titers of >1:400, meanwhile showing significant agglutination; 4) positive complement fixation test (CFT) with titers of >1:10, meanwhile showing significant agglutination; 5) positive serum tube agglutination test (SAT) with titers of >1:100. The exclusion criteria included 1) incomplete medical history data; 2) positive HIV antibody and AIDS; 3) patients who participated in other clinical trials during the same period; 4) pregnant and lactating women or women of childbearing age who are ready to conceive.

The primary outcome of this study was the treatment duration and outcome (cured, dead, or on treatment) within 2 years after treatment. Main secondary outcomes included results of serologic tests, and patient status (cured, dead or on-treatment) at day 42 or day 180 after

treatment initiation. This study had been registered on ClinicalTrials.gov Protocol Registration and Results System (NCT04020536).

Data Collection and Definitions

Electronic health records were collected with a medical record system designed for real-world studies provided by Huashan Hospital across 8 centers. Basic information on epidemiologic exposure history, symptoms and signs, complications and comorbidities, pathogenic diagnosis data, laboratory test data, and antibiotic therapy on the first medical visit were collected. Symptom, serology, examination, laboratory test, antibiotic therapy variation was collected at 14, 28, 42, 90, 180, 360, and 720 days after treatment initiation, until follow-up reached 2 years or lost. Clinical symptoms, treatment course and outcomes, etc. were recorded according to the hospital information system. Additional surveys on the telephone were performed on day 42, 90, 180, 365 or 730 if patients did not come to the hospital for regular medical visits.

Systemic involvement of brucellosis was diagnosed based on combination of symptoms, physical examination, and radiographic imaging according to the guidelines. Arthritis was diagnosed based on radiologic examination including x-ray, computed tomography (CT), or magnetic resonance imaging (MRI). Urogenital inflammation was diagnosed based on physical examination and ultrasound. Cardiac inflammation including endocarditis and myocarditis was diagnosed based on echocardiography, cardiac enzyme, electrocardiogram, and differentiated with other heart disease. Neurobrucellosis was diagnosed based on physical examination, cerebrospinal fluid tests, cranial radiologic findings, and differentiated with other possible pathogens. Early phase is defined as the natural history of brucellosis with symptom duration <180 days before patients received antibiotic therapy, while late phase is defined as duration >180 days. Acute cases are defined as patients who symptomatically recover within 6 months of treatment. Chronic brucellosis is defined as brucellosis-associated symptoms persisting over 6-month treatment according to expert consensus in China (*1*). Recurrence is defined as the

reappearance of symptoms, or new positive blood cultures after treatment completed and clinical manifestations disappeared (*2*).

Statistical Analysis

Continuous variables were expressed as median (interquartile range) and compared with the Mann-Whitney U test; categorical variables were expressed as number (%) and compared by χ^2 test or Fisher exact test. Symptoms, serology, and treatment variation was described with Kaplan-Meier curve and log-rank test. Propensity-score matching (PSM) was used to balance the difference between acute and chronic group, and risk factors were analyzed by logistic regression model. Two-tailed $p<0.05$ was considered statistically significant. Statistical analyses were performed using R software version 4.0.2. All figures were plotted by ggplot2 package.

References

1. Zhang W, Zhang Y. Chinese consensus guidelines for diagnosis and management of Brucellosis [cited 2020 Oct 24]. Chinese Journal of Infectious Diseases. 2017;35:705–10. https://kns.cnki.net/kcms/detail/detail.aspx?dbcode=CJFD&dbname=CJFDZHYX&filename=ZHCR201712001&v=sN5G45eVI%25mmd2Fat9QoWJOcHw1HZ2lAhahab4JxrG07mQPVlruZm7rIQrBZkc1821u9p

2. Ariza J, Gudiol F, Pallares R, Viladrich PF, Rufi G, Corredoira J, et al. Treatment of human brucellosis with doxycycline plus rifampin or doxycycline plus streptomycin. A randomized, double-blind study. Ann Intern Med. 1992;117:25–30. PubMed https://doi.org/10.7326/0003-4819-117-1-25

Appendix Table. Baseline characteristics between brucellosis diagnosed by culture and serum agglutination test*

Characteristic	Culture Positive SAT±, n = 380	Culture Negative SAT positive, n = 380	p value
Median age (IQR)	50.0 (41.0–58.0)	50.5 (41.0–58.0)	0.9558
Male	280 (73.7)	276 (72.6)	0.8060
Nationality Han	312 (82.1)	317 (83.4)	0.7009
Occupation			
Farmers	215 (56.6)	261 (68.7)	0.0003
Herdsmen	28 (7.4)	32 (8.4)	
Veterinarian	7 (1.8)	2 (0.5)	
Others	124 (32.6)	76 (20.0)	
Contact History			
Exposure to suspicious animals	357 (93.9)	277 (72.9)	<0.0001
Contact with brucellosis patients	277 (72.9)	256 (67.4)	0.1129
Residence in endemic area	238 (62.6)	173 (45.5)	<0.0001
Exposure to Brucella	16 (4.2)	14 (3.7)	0.8522
Symptoms			
Median duration, d (IQR)	30.0 (14.0–60.0)	30.0 (14.0–60.0)	0.7583
Fever	311 (81.8)	224 (58.9)	<0.0001
Sweating	177 (46.6)	95 (25.0)	<0.0001
Myalgia	77 (20.3)	76 (20.0)	0.9999
Arthralgia	251 (66.1)	251 (66.1)	0.9999
Poor Appetite	271 (71.3)	195 (51.3)	<0.0001
Hepatosplenomegaly	67 (17.6)	45 (11.8)	<0.0001
Headache	51 (13.4)	39 (10.3)	0.2169
Comorbidities			
Arthritis	111 (29.2)	119 (31.3)	0.5804
Urogenital inflammation	45 (11.8)	45 (11.8)	0.9999
Neurobrucellosis	8 (2.1)	10 (2.6)	0.8115
Cardiac inflammation	5 (1.3)	1 (0.3)	0.2188
Laboratory Tests, Mean ± SD			
C-reactive Protein (mg/dl)	34.5 ± 1.8	24.7 ± 1.7	0.0002
Procalcitonin (ng/ml)	0.2 ± 0.0	0.1 ± 0.0	0.1148
Erythrocyte Sedimentation Rate (mm/h)	45.6 ± 1.7	29.3 ± 1.4	0.0290

*Values are no. (%) except as indicated.

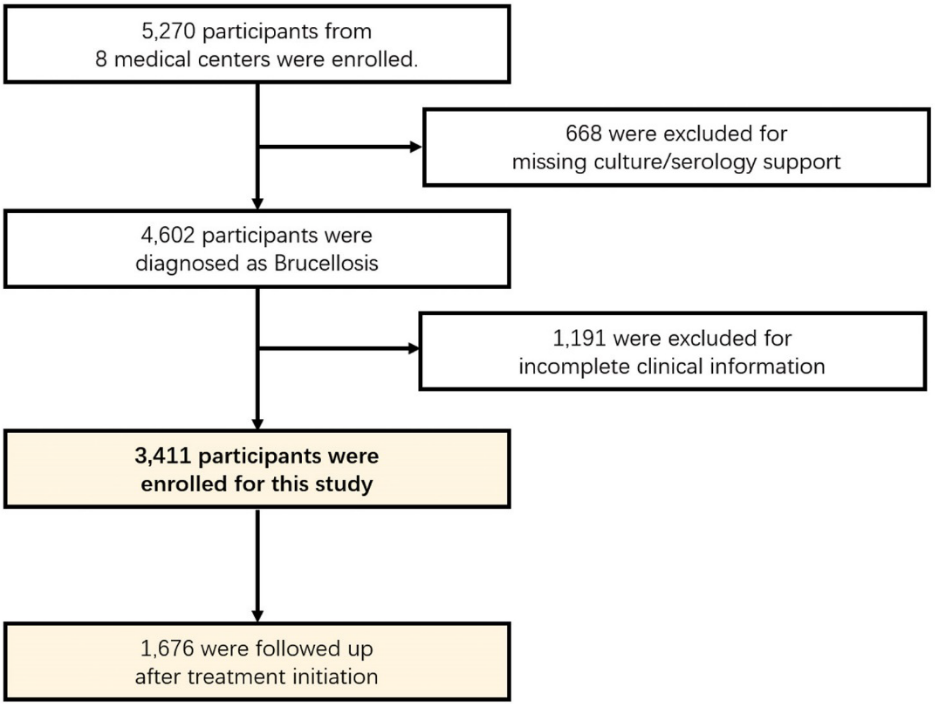

Appendix Figure 1. Flow chart of patient enrollment of brucellosis in 8 hospitals in China during 2014–2020

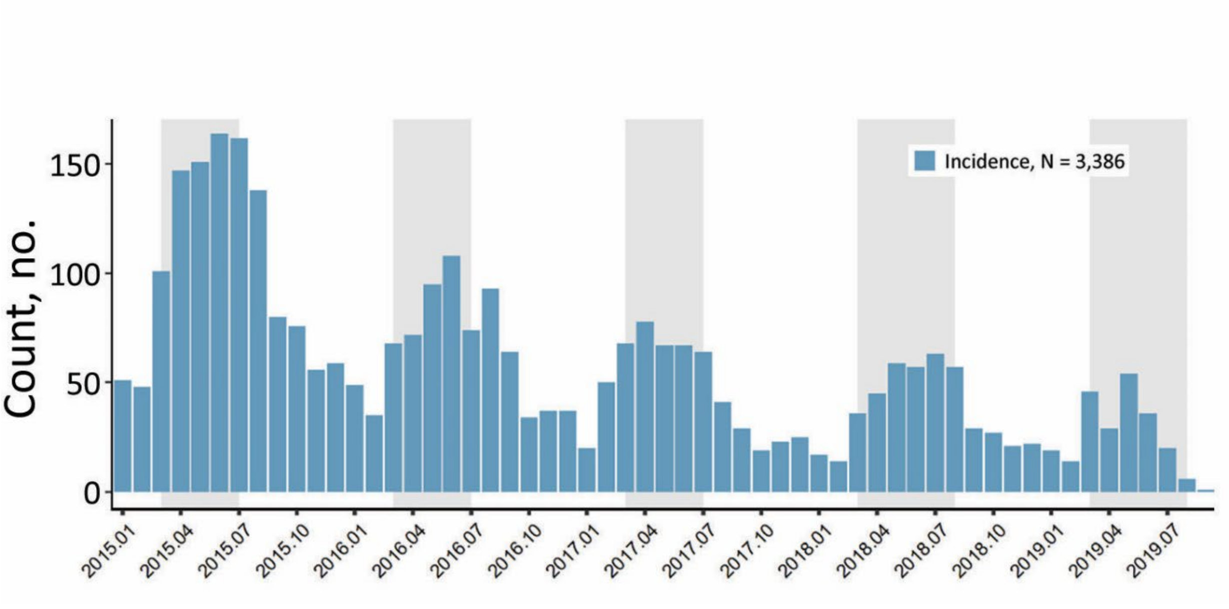

Appendix Figure 2. Number of patients diagnosed with brucellosis during 2014–2019. Of 3,411 patients, accurate diagnosis time was available for 3,222 patients.

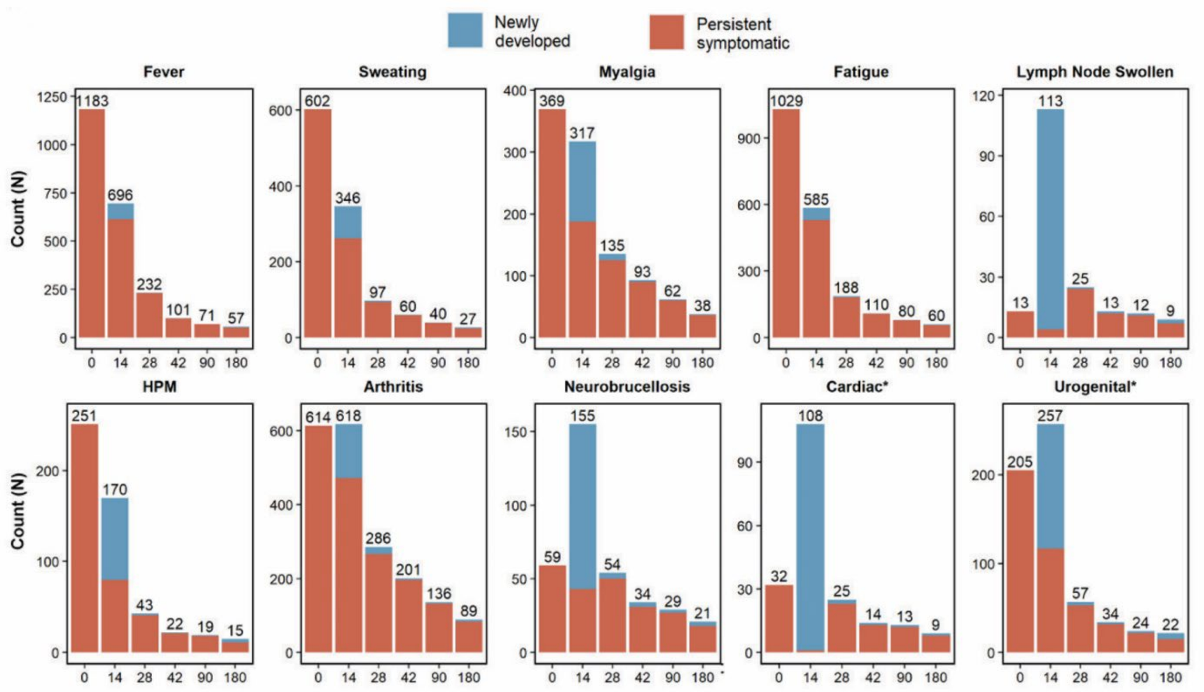

Appendix Figure 3. Dynamic symptom development after treatment initiation in case-patients with brucellosis, China, 2014–2020.

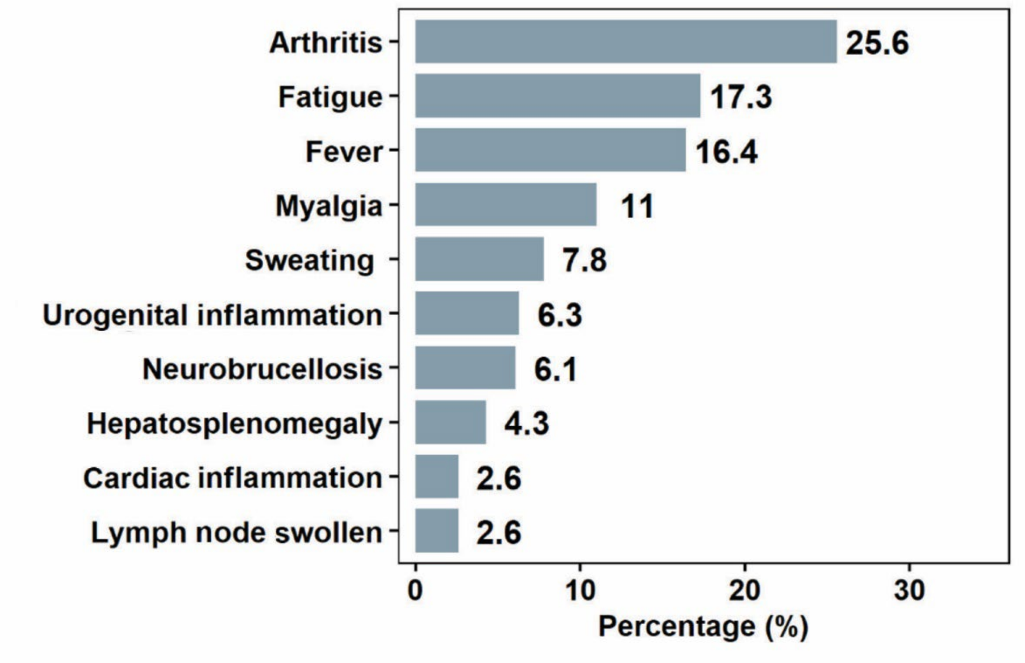

Appendix Figure 4. Symptom profiles present at chronic phase of chronic brucellosis, China, 2014–2020.
